# Photoelectrochemical H_2_ Evolution with a Hydrogenase Immobilized on a TiO_2_‐Protected Silicon Electrode

**DOI:** 10.1002/anie.201511822

**Published:** 2016-04-08

**Authors:** Chong‐Yong Lee, Hyun S. Park, Juan C. Fontecilla‐Camps, Erwin Reisner

**Affiliations:** ^1^ Department of ChemistryUniversity of CambridgeCambridgeCB2 1EWUK; ^2^ Metalloproteins Unit, Institut de Biologie Structurale, CEA, CNRSUniversité Grenoble Alpes38044GrenobleFrance; ^3^ ARC Centre of Excellence for Electromaterials ScienceIntelligent Polymer Research Institute/AIIM FacultyInnovation CampusUniversity of WollongongWollongongNSW2522Australia; ^4^ Fuel Cell Research CenterKorea Institute of Science and Technology (KIST)02792SeoulRepublic of Korea

**Keywords:** hydrogen evolution, hydrogenase, photoelectrochemistry, semiconductors, TiO_2_

## Abstract

The combination of enzymes with semiconductors enables the photoelectrochemical characterization of electron‐transfer processes at highly active and well‐defined catalytic sites on a light‐harvesting electrode surface. Herein, we report the integration of a hydrogenase on a TiO_2_‐coated p‐Si photocathode for the photo‐reduction of protons to H_2_. The immobilized hydrogenase exhibits activity on Si attributable to a bifunctional TiO_2_ layer, which protects the Si electrode from oxidation and acts as a biocompatible support layer for the productive adsorption of the enzyme. The p‐Si|TiO_2_|hydrogenase photocathode displays visible‐light driven production of H_2_ at an energy‐storing, positive electrochemical potential and an essentially quantitative faradaic efficiency. We have thus established a widely applicable platform to wire redox enzymes in an active configuration on a p‐type semiconductor photocathode through the engineering of the enzyme–materials interface.

Hydrogenases are enzymes that catalyze the reversible reduction of protons to H_2_ at record rates, and have therefore attracted much attention as a noble‐metal‐free benchmark catalyst in the fuel‐forming reaction of water splitting.^1^ Research into hydrogenases has resulted in an in‐depth understanding of catalytic function and inspired the design of both structural^2^ and functional^3^ H_2_ evolution catalysts.

Hydrogenases have also emerged recently as model electrocatalysts in photocatalytic H_2_ generation schemes, in which the enzyme is coupled to light‐harvesters, such as carbon nitride, dye‐sensitized TiO_2_, Cd‐based quantum dots, and organic dyes such as Eosin Y.^4^ In these systems, the hydrogenase can efficiently collect photo‐excited electrons via its intraprotein FeS cluster relays and deliver them to the embedded active site for H_2_ generation at benchmark turnover rates. However, a sacrificial electron donor is required in all of these systems, which limits the utility of the overall redox chemistry and prevents solar water splitting in these systems. Previously, a hydrogenase has also been adsorbed onto a semiconductor electrode composed of n‐type CdS‐ and TiO_2_‐based materials.^5^ In this case, light‐driven H_2_ evolution was only possible at a potential more negative than the thermodynamic equilibrium potential in the dark and consequently the storage of light energy in H_2_ could not be demonstrated.

The [NiFeSe]‐hydrogenase isolated from *Desulfomicrobium baculatum* (*Dmb*) is a particularly suitable H_2_ evolution catalyst for water splitting as it displays a strong bias towards H_2_ evolution in the presence of O_2_.^6^, ^7^, ^8^ As such, we were able to demonstrate quantitative water splitting and the net storage of light energy as H_2_ using this hydrogenase, wired to a photosystem II‐based photoanode, in a photoelectrochemical cell.^9^ However, this system relies on the hydrogenase being adsorbed onto a hierarchical indium–tin oxide electrode, which requires an applied bias to perform proton reduction.^9^ Bias‐free water splitting may be achieved by the incorporation of the hydrogenase on a suitable p‐type semiconducting electrode and complementing this to a suitable photoanode. However, immobilization of functional enzymes onto a p‐type semiconductor material remains a major challenge,^10^ largely owing to the intrinsic instability of the available materials and their fragile interface with biological materials. Photoelectrocatalytic H_2_ production with a hydrogenase on a p‐type semiconductor electrode is unknown.

The challenge is therefore to find a suitable p‐type photocathode material that is stable, allows a favorable interaction with the hydrogenase and has suitable band levels to enable visible‐light‐driven proton reduction. p‐Type silicon has been widely regarded as one of the most promising photocathode materials as it has a small band gap of 1.1 eV with a conduction‐band edge position of approximately −0.6 V versus the standard hydrogen electrode (SHE).^11^ However, enzyme integration with a bare Si surface is limited by the electrode instability in aqueous solution owing to the formation of an insulating SiO_2_ layer. Herein, we present a p‐Si|TiO_2_|hydrogenase photocathode, which contains an amorphous TiO_2_ protection layer between the semiconductor and the enzyme. Amorphous and thin TiO_2_ is a conductor on Si and known to protect the Si surface.^12^ In addition, we show that biocompatibility of the amorphous TiO_2_ film enables productive adsorption of the hydrogenase. The engineered interface in this semiconductor–enzyme electrode allows us to assemble a hydrogenase‐based photocathode capable of storing light energy (i.e., showing light‐induced cathodic response at a potential more positive than *E* ^o^′(H^+^/H_2_)).

Figure 1 summarizes the key features of the proposed *p*‐Si|TiO_2_|hydrogenase photocathode: photoexcitation of Si by visible light generates low‐potential electrons in the semiconductor conduction band, which are transferred to the immobilized hydrogenase via the thin layer of TiO_2_. The electrons enter the enzyme through the distal FeS cluster and reach the [NiFeSe] active site, where the reduction of protons to H_2_ occurs.^7^ This mechanistic consideration is facilitated by the TiO_2_ conduction‐band potential at approximately −0.6 V versus SHE, which is located between the conduction band of Si and the H^+^/H_2_ reduction potential. Thus, TiO_2_ can be considered as a conductor under the reducing conditions provided by the excited Si.4b,4c,4e, 12b,12e

**Figure 1:**
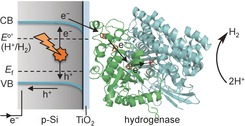
Schematic representation of the p‐Si|TiO_2_|hydrogenase photocathode. The amorphous TiO_2_ acts as a bifunctional interface layer as it protects Si from the formation of an insulating oxide coating and provides a biocompatible surface to facilitate the adsorption of *Dmb* [NiFeSe]‐hydrogenase. VB=valence band, CB=conduction band, *E*_f_=Fermi level.

First, we examined the interaction of *Dmb* [NiFeSe]‐hydrogenase with amorphous TiO_2_ coated on a fluoride‐doped tin oxide (FTO) electrode. An amorphous TiO_2_ layer was prepared by drop‐casting TiCl_4_ in toluene onto an FTO‐coated glass substrate (3 μL of 2 mm solution per cm^2^) and hydrolysis in air, and this step was repeated twice resulting in a film thickness of approximately 500 nm (Figure S1 a in the Supporting Information). The formation of TiO_2_ was confirmed by X‐ray photoelectron spectroscopy (XPS) and by energy dispersive X‐ray spectroscopy (EDX) elemental analysis (Figures S1 b and S2). This method to generate an amorphous TiO_2_ coating through solution processing at room temperature is simple and widely applicable,^13^ which is in contrast to conventionally employed atomic‐layer deposition or sputtering technologies to produce TiO_2_ films.12a,12c The FTO|TiO_2_ surface was subsequently rinsed with water, the enzyme (3 μL of 8 μm solution per cm^2^) was drop‐cast onto the TiO_2_ surface and the enzyme‐modified electrode rinsed with the electrolyte solution prior electrochemical measurements (50 mm MES (2‐(N‐morpholino)ethanesulfonic acid), at pH 6.0).

Figure 2 shows the protein film voltammogram^14^ of the FTO|TiO_2_|hydrogenase electrode in a three‐electrode configuration, with a Ag/AgCl and a Pt wire as reference and counter electrodes, respectively (see Supporting Information for details). The catalytic current is associated to the activity of the immobilized hydrogenase,1b, 14 and the voltammograms display the characteristic features of a hydrogenase wired onto the electrode. Proton reduction and H_2_ oxidation currents can be observed with a small overpotential. In the absence of an immobilized enzyme, no catalytic response is observed and only TiO_2_‐characteristic charging currents can be seen in the cyclic voltammogram (Figure S3).

**Figure 2:**
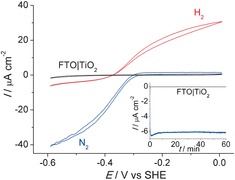
Protein film electrochemistry with FTO|TiO_2_|hydrogenase. The voltammograms were recorded for a stirred sample, under an atmosphere of N_2_ (blue trace) and 1 bar H_2_ (red trace) at a scan rate of 10 mV s^−1^. A control experiment (black trace) in the absence of enzyme is also shown. The inset shows the CPE trace at *E*_appl_=−0.35 V versus SHE under N_2_ for FTO|TiO_2_ (black) and FTO|TiO_2_|hydrogenase (blue). All experiments were performed in MES (50 mm) electrolyte solution with a Ag/AgCl reference and Pt counter electrode at pH 6.0 at 20±2 °C.

Controlled potential electrolysis (CPE) was performed to determine the stability and Faradaic yield of H_2_ evolution. The hydrogenase was adsorbed onto amorphous TiO_2_, and an applied potential (*E*_appl_) of −0.35 V versus SHE was applied, which corresponds to 0 V versus the reverse hydrogen electrode (RHE) at pH 6.0.^15^ After 1 h CPE under a N_2_ atmosphere, a charge of 18 mC had passed and 90±5 nmol of H_2_ accumulated, which corresponds to a Faradaic yield of 96±6 %. These results demonstrate that amorphous TiO_2_ acts as a suitable interfacial layer for the immobilization of electroactive *Dmb* [NiFeSe]‐hydrogenase; this interaction is believed to occur at the surface‐exposed glutamate and aspartate residues in close proximity to the distal Fe‐S cluster.1f, 6, 7 Protein film voltammetry with hydrogenases on crystalline TiO_2_ had been reported previously,4b, 16 but the preparation of these metal oxide films required high‐temperature annealing, which is not compatible with the integration on a silicon electrode (see below).

The amorphous TiO_2_ layer (3 μL of 2 mm TiCl_4_ in toluene solution per cm^2^) was subsequently applied on a pretreated p‐Si with an atomically flat H‐terminated surface. This step was repeated twice, followed by rinsing with water and adsorption of the enzyme (3 μL of 8 μm solution per cm^2^) using the same method as on the FTO‐coated glass electrodes (see Supporting Information for details). The protein film photoelectrochemical response under chopped white‐light illumination (10 mW cm^−2^) recorded under an N_2_ atmosphere is summarized in Figures 3 a and Figure S4. The p‐Si|TiO_2_|hydrogenase photocathode showed an onset photocurrent of approximately −0.1 V versus SHE (i.e., approximately 0.25 V more positive than *E* ^0^′(H^+^/H_2_)), thereby demonstrating the capability to operate at a thermodynamic underpotential with this electrode. In control experiments, the linear sweep voltammograms show the expected low photoactivity of the TiO_2_‐free p‐Si electrodes (corresponding to bare p‐Si and p‐Si|hydrogenase, where the enzyme was adsorbed on bare p‐Si); a result of the fast formation of an insulating SiO_2_ layer on the semiconductor surface.^17^ The enzyme‐free p‐Si|TiO_2_ electrode displays a photo‐response in the cathodic region (presumably owing to charging of the TiO_2_ conduction band), but the current density is significantly lower at less‐negative potentials than that of the p‐Si|TiO_2_|hydrogenase, indicating significantly faster H_2_ evolution kinetics with the enzyme–semiconductor hybrid system. No photocurrent response was observed with an amorphous TiO_2_ film on FTO‐coated glass (Figure S5).

**Figure 3:**
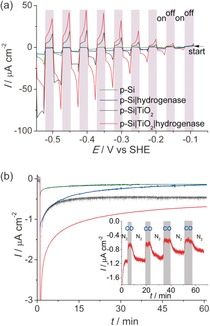
Protein film photoelectrochemistry with p‐Si|TiO_2_|hydrogenase. a) Photoresponse under chopped irradiation (10 mW cm^−2^; gray shading indicates response in the dark) performed at a scan rate of 10 mV s^−1^ under a N_2_ atmosphere. b) CPE at *E*_appl_=−0.35 V versus SHE (pH 6.0) during irradiation under a N_2_ atmosphere; the color labeling of the traces from (a) applies also to the CPE traces in (b). Inset shows the effect of 10 % CO injections (highlighted in gray) on the photocurrent response of p‐Si|TiO_2_|hydrogenase at *E*_appl_=−0.35 V versus SHE, followed by flushing with 100 % N_2_. All experiments were performed in MES (50 mm) electrolyte solution with a Ag/AgCl reference and Pt counter electrode at pH 6.0 at 20±2 °C under white‐light illumination with an intensity of 10 mW cm^−2^.

Thus, the p‐Si|TiO_2_|hydrogenase electrode exhibits an enhanced photoresponse compared to the TiO_2_‐ and hydrogenase‐free p‐Si electrodes, in particular at less‐negative potentials, where energy from irradiation can be stored. These higher photocurrents are due to the efficient collection of conduction‐band electrons by the electroactive enzyme. This work demonstrates the integration of a hydrogenase on Si and optimizations to achieve higher photocurrent densities are currently in progress. The photocurrent density is currently limited by the sub‐optimal integration of the hydrogenase in the TiO_2_ protection layer (i.e., the absence of a porous morphology for high protein loading) and the low light intensity (10 mW cm^−2^) employed in this study.

CPE was performed to confirm photoelectrochemical H_2_ formation and to study the robustness of p‐Si|TiO_2_|hydrogenase. The electrolysis experiments were performed at *E*_appl_=−0.35 V versus SHE (0 V vs. RHE) under N_2_ and in the dark for 60 s, followed by white‐light illumination (10 mW cm^−2^) for 1 h (Figure 3 b). An initial decrease in the photocurrent was observed, followed by stabilization of the photoresponse with p‐Si|TiO_2_|hydrogenase. After 1 h, a charge of 5.1±0.2 mC had passed and 25±2 nmol of H_2_ were detected, which corresponds to a Faradaic yield of 95±6 %. In the absence of hydrogenase or TiO_2_, substantially lower photocurrents were observed and we were unable to reliably detect H_2_ in the control experiments with p‐Si|TiO_2_ and p‐Si|hydrogenase.

Finally, further control experiments were performed to unambiguously demonstrate that the enzyme was indeed the active catalyst on the electrode. The inset in Figure 3 b shows the current–time profile at *E*_appl_=−0.35 V versus SHE under an alternating N_2_ and 10 % CO in N_2_ atmosphere, and during white‐light illumination. CO was selected as it is a reversible inhibitor of the *Dmb* [NiFeSe]‐hydrogenase active site.^7^ The photocurrent was substantially reduced upon addition of CO. Removal of CO by N_2_ purging of the electrolyte solution shows the recovery of the photocurrent. This response is consistent with the characteristic of reversible inhibition of the hydrogenase and can be repeated for several cycles. Some photocurrent remained even in the presence of CO, which is attributed to the background photocurrent of the amorphous TiO_2_ layer (see above).

In summary, we have established an easily applicable methodology for interfacing redox enzymes with p‐type semiconductor electrodes to promote light‐driven reductive reactions. In doing so, we have also extended the use of TiO_2_ protection layers from anchoring synthetic catalysts to biological catalysts. Specifically, we demonstrate with this interfacial engineering approach that a hydrogenase can be coupled to a p‐type silicon electrode, which allows for photocatalytic H_2_ generation with a hydrogenase on a semiconductor in the absence of a sacrificial agent and electrochemical overpotential. This work takes us closer to the construction of semi‐biological photoelectrochemical systems and the assembly of an all‐enzyme driven, bias‐free photoelectrochemical water‐splitting cell with the O_2_‐tolerant *Dmb* [NiFeSe]‐hydrogenase. Work is currently in progress to enhance the performance of the photocathode through rational materials design for an optimized immobilization of the hydrogenase.

## Supporting information

As a service to our authors and readers, this journal provides supporting information supplied by the authors. Such materials are peer reviewed and may be re‐organized for online delivery, but are not copy‐edited or typeset. Technical support issues arising from supporting information (other than missing files) should be addressed to the authors.

Supplementary
