# Supplementary material for: Photoelectrochemical H2 Evolution with a Hydrogenase Immobilized on a TiO2‐Protected Silicon Electrode
Source: Angew Chem Int Ed Engl. 2016 Apr 8;55(20):5971–4. doi: 10.1002/anie.201511822 (PMC4981910; doi:10.1002/anie.201511822)
Supplement: Supplementary file 1 — Supplementary [file ANIE-55-5971-s001.pdf]

Angewandte Chemie
Eine Zeitschrift der Gesellschaft Deutscher Chemiker

Supporting Information

Photoelectrochemical H_2 Evolution with a Hydrogenase Immobilized on a TiO_2-Protected Silicon Electrode

*Chong-Yong Lee, Hyun S. Park, Juan C. Fontecilla-Camps, and Erwin Reisner**

anie_201511822_sm_miscellaneous_information.pdf

Experimental Section.

Chemicals. All chemicals were purchased from commercial suppliers and used without further purification unless otherwise noted. Reagents for the analytical part of the work were of the highest available purity. Milli-Q water (18 MΩ cm) was used for the electrochemical experiments. Silver loaded epoxy adhesive (RS Components Ltd.) was used to make electrical contact to the electrodes. Fluorine-doped tin oxide (FTO) glass (TEC 7, ~7 ohm sq^{-1}, Sigma-Aldrich) was used.

Isolation and purification of [NiFeSe]-hydrogenase. [NiFeSe]-hydrogenase from *Desulfomicrobium baculatum* was purified using a previously published method (E. C. Hatchikian, M. Bruschi, J. Legall, *Biochem. Biophys. Res,* **1978**, *82*, 451–461). The pure enzyme was dialyzed against 20 mM Tris/HCl at pH 7.6. The enzyme integrity was verified spectrophotometrically at λ = 604 nm by measuring its specific activity for H_2 oxidation with an aliquot of the hydrogenase under H_2 in the presence of methyl viologen (1 mM) for 30 min at 30 °C. The preparation has a specific activity of 2115 μmol H_2 min^{-1} mg^{-1}, and the stock enzyme solution was diluted with 20 mM Tris/HCl buffer in an anaerobic glovebox to obtain a concentration of 8 μM before adsorption on the electrodes.

Preparation of p-Si electrodes. p-Si photocathodes were made from commercial boron-doped Si wafers (University Wafers; resistivity of 1-10 ohm cm; (100) single-side polished). The wafers were cut into small pieces (~ 0.6 to ~1.2 cm^2) using a diamond glass cutter. The electric contact was formed using a Ga:In eutectic solution (99.99%; Sigma-Aldrich) and a copper wire (RS Components Ltd) covered with a conductive silver epoxy resin (RS Components Ltd). The geometric area of photocathodes was defined using a Teflon tape enclosure. Before the deposition of TiO_2 and hydrogenase, native oxide layers and inorganic/organic impurities on the silicon surface were removed by sequential treatments using H_2O_2 (30 wt.% in H_2O; Sigma-Aldrich), H_2SO_4 (95-98%; Sigma-Aldrich), and HF (65%; Merck

Millipore) etchants for 1 min at each step. This treatment was followed by etching in 40% NH_4F for at least 10 min to obtain atomic flat H-terminated surfaces, that was followed by rinsing with miliQ water.

Preparation of p-Si|TiO_2 and FTO|TiO_2. A solution of 3 μL (per cm^2) of titanium tetrachloride (Sigma Aldrich, 2.0 mM in dry toluene) was dropcast on the FTO-coated glass substrates or the etched p-Si surface. A thin oxide layer was formed rapidly in air, and this step was repeated twice unless otherwhise noted. After the final deposition, the electrode was rinsed with water to remove potentially unreacted $TiCl_4$ and the by-product, HCl.

Preparation of p-Si|hydrogenase or p-Si|TiO_2|hydrogenase electrodes. The hydrogenase stock solution (8 μM, 20 mM Tris/HCl buffer, pH 7.0) was stored in small volume vials at –30 °C inside an anaerobic glovebox, and was thawed immediately before use. Then, 3 μL per cm^2 of this hydrogenase solution was dropcast onto the etched p-Si or p-Si|TiO_2. The p-Si|hydrogenase or p-Si|TiO_2|hydrogenase electrode was rinsed with the electrolyte solution prior to electrochemical studies to remove all unbound enzyme from the electrode surface.

Electrochemical and photoelectrochemical measurements. Protein film voltammetry was performed in a three-electrode configuration with a Ag/AgCl (3M NaCl) (Bioanalytical Sys.) reference and a platinum counter electrode. A CompactStat.e potentiostat (Ivium Technologyies B.V.) was used for the electrochemical experiments. All redox potentials are quoted against the standard hydrogen electrode (SHE) and the potentials were obtained by using the following correction factor: $E_{\mathrm{SHE}} = E_{Ag/AgCl} + 0.209$ V. All experiments were carried out in an anaerobic MBraun glovebox with an O_2 concentration of less than 1 ppm. An IVIUM modulight LED module with white light of 10 mW cm^{-2} was employed as light source.

Physical Characterization. The surface morphology of the TiO_2 electrode was analyzed by a scanning electron microscopy (SEM, Philips XL30). Elemental analysis was performed by a Bruker X-Flash 4010 energy dispersive X-ray (EDX) detector. XPS was performed on a Thermo Scientific ESCALAB 250Xi XPS Microprobe utilizing a monochromatic Al-Kα X-ray source. The amount of H_2 generated in the headspace of the electrochemical cell was detected and quantified by gas chromatography (GC). An Agilent 7890A Series gas chromatograph equipped with a 5 Å molecular sieve column and a thermal conductivity detector was employed. Approximately 40 μL aliquots of the headspace gas were taken from the airtight cell for GC analysis.

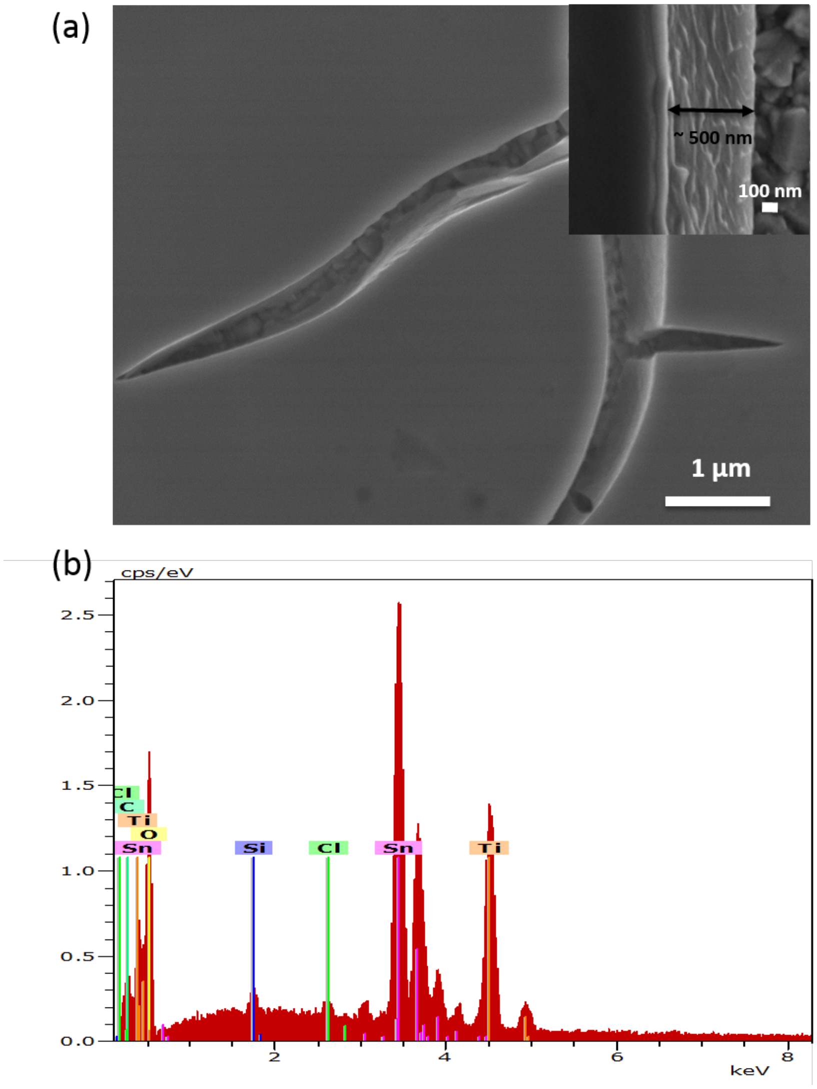

Figure S1. SEM (a) top view image, with inset showing a cross-sectional image, and (b) EDX mapping of amorphous TiO_2 layer prepared by drop-casting $TiCl_4$ in toluene onto a FTO-coated glass substrate rinsed with water and dried prior measurement.

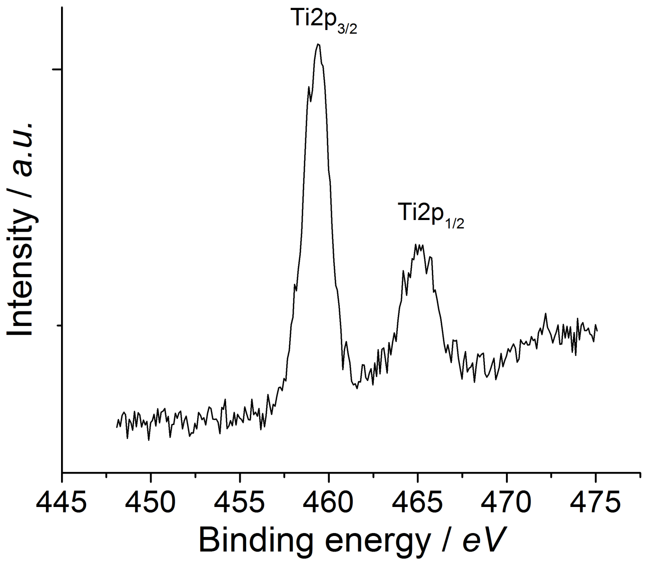

Figure S2. XPS spectrum of the Ti2p peaks of the amorphous TiO_2 sample prepared by drop-casting $TiCl_4$ in toluene onto an FTO-coated glass substrate (rinsed with water and dried prior measurement). $Ti2p_{3/2}$ and $Ti2p_{1/2}$ peaks are at 459.26 eV and 465.03 eV, respectively. The $Ti2p_{3/2}$ value is in agreement with the literature TiO_2 values of 459.36 eV (see B. Erdem, R. A. Hunsicker, G. W. Simmons, E. D. Sudol, V. L. Dimonie, M. S. El-Aasser, *Langmuir* 2001, *17,* 2664-2669) and 459.1 eV (P. Stefanov, M. Shipochka, P. Stefchev, Z. Raicheva, V. Lazarova, L. Spassov, *J. Phys.: Conf. Ser.*, 2008, 100, 012039). The observed peak positions, with the doublet separation between the $2p_{1/2}$ and $2p_{3/2}$ peaks of ~5.8eV, further confirms the presence of TiO_2.

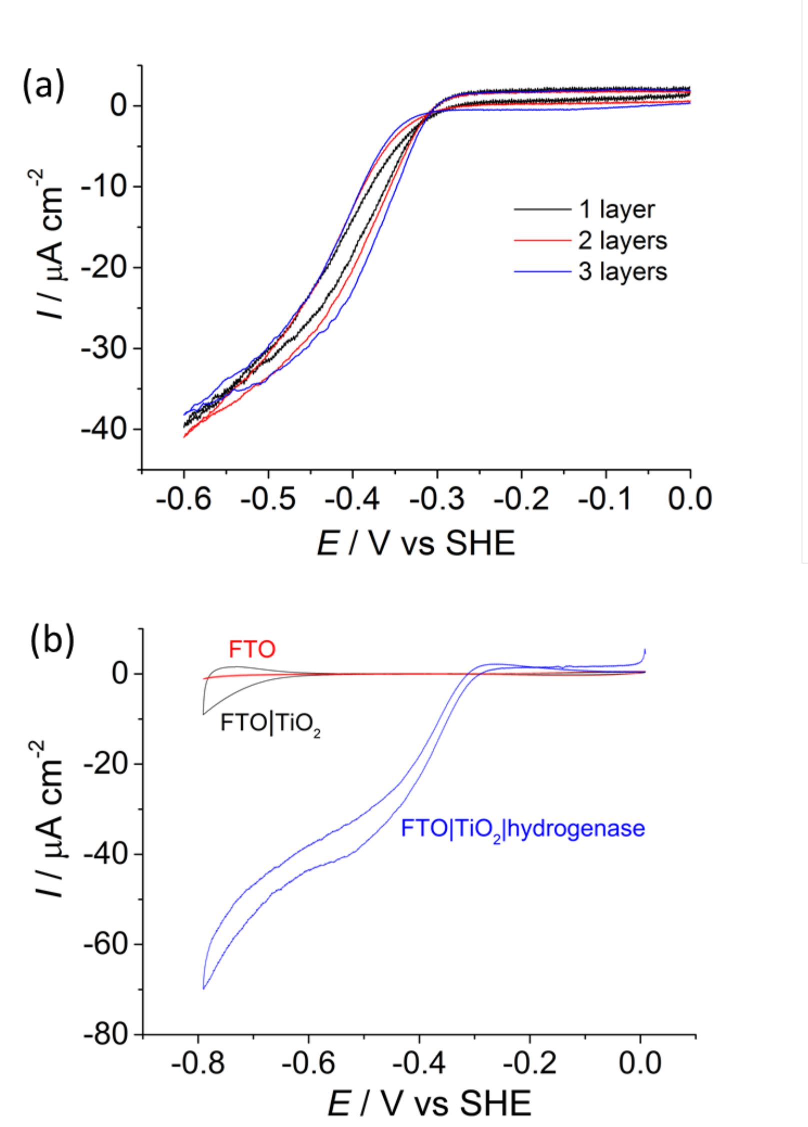

Figure S3. Cyclic voltammograms of (a) FTO|TiO_2|hydrogenase with different layers of deposited $TiCl_4$ (see main text for details) and (b) FTO|TiO_2|hydrogenase (blue trace), FTO|TiO_2 (black trace) and bare FTO-coated glass (red trace). Voltammograms were recorded at a scan rate of 10 mV s^{-1} in a MES (50 mM) electrolyte solution at pH 6.0 and room temperature. Noted that 2 layers of $TiCl_4$ were deposited in (b).

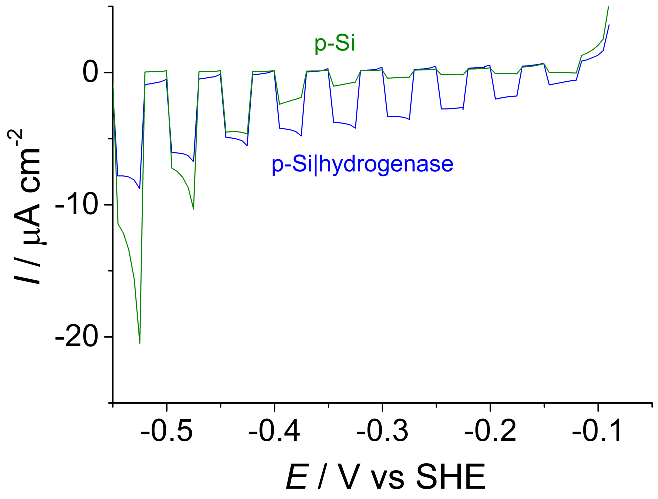

Figure S4. Chopped photoresponses of bare p-Si (green trace) and p-Si|hydrogenase (blue trace) performed at a scan rate of 10 mV s^{-1} in MES (50 mM) electrolyte solution at pH 6.0.

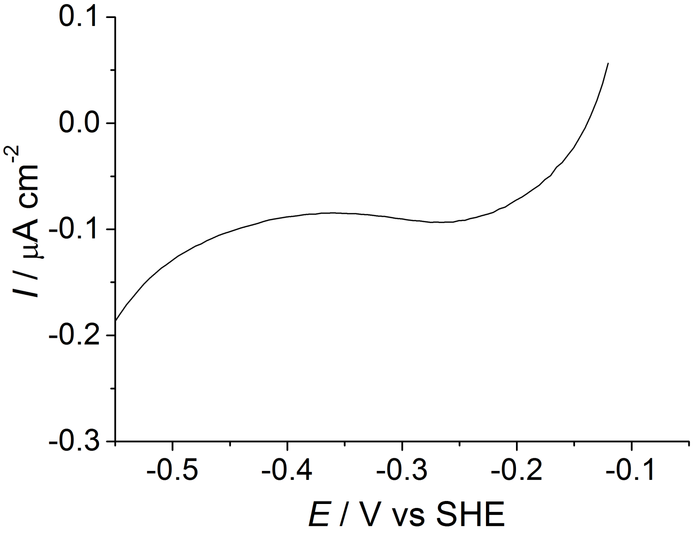

Figure S5. Control experiment with linear sweep voltammogram of FTO|TiO_2 under chopped light performed at a scan rate of 10 mV s^{-1} in MES (50 mM) electrolyte solution at pH 6.0. The chopped light was followed time interval as in Figure S4 and no photoresponse was observed on this electrode.

End of Supporting Information
